# Synthetic oligonucleotide antigens modified with locked nucleic acids detect disease specific antibodies

**DOI:** 10.1038/srep35827

**Published:** 2016-10-24

**Authors:** Simone V. Samuelsen, Ilia A. Solov’yov, Imelda M. Balboni, Elizabeth Mellins, Christoffer Tandrup Nielsen, Niels H. H. Heegaard, Kira Astakhova

**Affiliations:** 1 Department of Physics, Chemistry and Pharmacy, University of Southern Denmark, Odense M, 5230, Denmark; 2 Department of Pediatrics, Division of Allergy, Immunology and Rheumatology, Stanford University School of Medicine, Palo Alto, CA, 94304, USA; 3 Department of Pediatrics, Program in Immunology, Stanford University School of Medicine, Stanford, CA, 94305, USA; 4 Department of Autoimmunology and Biomarkers, Statens Serum Institute, DK-2300 Copenhagen S, Denmark; 5 Copenhagen Lupus and Vasculitis Clinic, Centre for Rheumatology and Spine Diseases, Rigshospitalet, Copenhagen University Hospital, Copenhagen, 2100, Denmark; 6 Department of Clinical Biochemistry and Pharmacology, Odense University Hospital, University of Southern Denmark, DK-5000 Odense C, Denmark

## Abstract

New techniques to detect and quantify antibodies to nucleic acids would provide a significant advance over current methods, which often lack specificity. We investigate the potential of novel antigens containing locked nucleic acids (LNAs) as targets for antibodies. Particularly, employing molecular dynamics we predict optimal nucleotide composition for targeting DNA-binding antibodies. As a proof of concept, we address a problem of detecting anti-DNA antibodies that are characteristic of systemic lupus erythematosus, a chronic autoimmune disease with multiple manifestations. We test the best oligonucleotide binders in surface plasmon resonance studies to analyze binding and kinetic aspects of interactions between antigens and target DNA. These DNA and LNA/DNA sequences showed improved binding in enzyme-linked immunosorbent assay using human samples of pediatric lupus patients. Our results suggest that the novel method is a promising tool to create antigens for research and point-of-care monitoring of anti-DNA antibodies.

Since their discovery in 1940s, human antibodies against nucleic acids have become ubiquitous as a tool in diagnostics and studies of human diseases^1^. This is the case in, for example, systemic lupus erythematosus (SLE)^2^. SLE is a systemic autoimmune disorder, potentially causing damage to virtually any organ in the body. The cause of SLE is not fully understood^3^, but anti-DNA antibodies often play a crucial role by triggering disease manifestations via antibody-DNA complex deposition, and are useful for the diagnosis of SLE^4^. However, as knowledge on antibodies to nucleic acids has increased, there has been a growing focus on their specificity. Ever-increasing results on anti-DNAs necessitate reliable tools for their specific detection and sub-typing^2^,^4^,^5^.

Three current laboratory methods are commonly used for the determination and quantification of anti-DNA: Crithidia luciliae indirect immunofluorescence (IIF), enzyme-linked immunosorbent assay (ELISA), and radio-immunoassay (RIA-the Farr assay)^2^. Comparison of these methods is limited, because of the great differences in methodologies (see Supplementary Fig. S1 for schematic representation of the assays). All assays thus require careful validation to determine robustness of antibody detection^5^. Although, the assays are performed under equilibrium conditions, unfortunately they are unable to provide any information on quantitative binding characteristics. Moreover, currently applied heterogeneous and unstable natural DNA antigens often result in poor reproducibility and low specificity of the blood tests; around 5% of healthy persons give a weak positive result, even though they are not suffering from SLE^2^. Detected anti-DNA antibodies also cross-react on other antigens (Supplementary Table S1).

Structural information on DNA‒antibody interaction is still rather limited. Moreover, as exclusively DNA of unknown sequence is used as antigen in the aforementioned assays, anti-DNA antibodies are classified only as targeting either single-stranded (ss) or double-stranded (ds) DNA^6^. Most people suffering from SLE have anti-dsDNA antibodies, whereas ssDNA binding antibodies are not specific to SLE and may also be found in people without disease^7^. It is also known that anti-dsDNA antibodies in sera may be any of the three isotypes (IgG, IgM, and IgA), but mostly IgG, and these are often present years before the onset of clinical symptoms^2^,^6^.

In general, DNA binds to antibodies through several types of interactions, particularly hydrogen bonds, van der Waals and electrostatic forces are important (Supplementary Table S2)^8^. Moreover, the hydrophobic contacts, together with the ion dipole bonds, contribute in a major way to the stability of protein-nucleic acid complexes, whereas hydrogen bonds with base edges are important for the specificity^9^. Recently, a conserved structural element which can be used for recognition of ssDNA has been identified, the ssDNA-Antibody Recognition Module (D-ARM). The D-ARM consists of a tyrosine residue that stacks with the base, and a glycine residue that forms a hydrogen bond with the base. Y. An *et al*.^10^ showed that the monoclonal antibody ED-10^11^ interacts with two adjacent nucleotides in its binding site and favours dTdC over other nucleotides. The second nucleotide, cytidine, is located in the D-ARM and engages in π-stacking interactions with a group of amino acids, i.e. with Y32, is perpendicular to W95 and forms hydrogen bonds with G91 and G98. Figure 1 shows DNA binding to the ED-10 antibody.

The number of experimentally resolved biomolecular structures is constantly increasing, with new sequences and atomic resolution structures deposited in databases. Structures yield static information that can be viewed with molecular graphics software, such as the molecular design and analysis program Visual Molecular Dynamics (VMD)^12^. Structures are also essential for dynamic studies that can provide insights into functions and mechanisms. For example, using the Nanoscale Molecular Dynamics Program (NAMD)^13^, it is possible to access dynamic information extrapolated from structures and study the interaction between two large biomolecules through molecular dynamics simulations.

Synthetic oligonucleotides may be produced with high purity, good specificity and affinity, and provide well-controlled chemical structures, which make them a promising tool for diagnostics and studies of autoimmune diseases where aberrant anti-DNA immunoreactivity occurs^14^. Rational design and the incorporation of modified nucleotides into oligonucleotides contribute to improvements, such as increased duplex stability and improved specificity of targeting^15^. One example of a modified nucleotide with promising properties is the locked nucleic acid (LNA). In 1998 Koshkin^16^ and Obika^17^ independently introduced LNA, being a structural modification of a standard nucleotide comprising a 2′-O, 4′-C methylene bridge (Fig. 1b compared to Fig. 1a), which locks the furanose ring in a C3′-endo conformation and has been found to increase the binding affinity towards complementary DNA. The thermal duplex stability is increased by 2–8 °C per incorporated LNA monomer, compared to unmodified duplexes, without compromising the base pairing selectivity^18^. Moreover, Jorgensen *et al*. demonstrated the unique selectivity and sensitivity of synthetic oligonucleotides containing LNA in the recognition of nucleic acid targets and antibodies against double-stranded DNA^19^.

Here, we investigate the possibility of using novel LNA/DNA oligonucleotides as antigens in the detection and study of antibodies to double stranded DNA (a-dsDNAs). The interaction between dsDNA and the model antibody ED-10 was first investigated computationally to determine which nucleobases give the best interaction and whether locking the nucleotides optimizes the binding (Fig. 1). One advantage of optimizing the binding between the antigen and specific antibody, in the case of monoclonal antibody ED-10, is that the risk of cross-reactivity is decreased and fewer false positive results are obtained. Successful LNA/DNA candidates were then tested in surface plasmon resonance (SPR) studies, which revealed their binding affinity to the target antibodies. Finally, we used LNA/DNA molecules to detect antibodies in pediatric lupus patients using ELISA assay.

## Results

### Oligonucleotide design and simulations

In recent work, we demonstrated that 21mer synthetic DNA/LNA molecules are efficient for binding monoclonal a-dsDNA antibodies and do not interact non-specifically with other proteins and antibodies^19^. Here, we hypothesized that synthetic nucleotides containing particular structural elements would provide optimized antigen(s) for detection of anti-DNA antibodies. We considered four variables: nucleotide composition, sequence polarity, modification with LNA and length of the synthetic oligonucleotides. Existing work on ED-10 monoclonal antibody argues that only two nucleotides are sufficient for the interaction when 5′-dTdC-3′ sequence is used as antigen^10^. In this interaction, nucleobases bind to the amino acids of ED10 and a hydrogen bond also is formed. Based on these results, we build our initial DNA strands as repeats of dinucleotides, starting with dTdC and then performing the mutations of the nucleotides within this repeat T → A and C  →T. As a control, we included mixmer (ATCG) sequence SEQ7 into the study; the sequence was adopted from our past work^19^.

As a next step, all the sequences were modified in order to avoid self-complementarity and hairpin formation. This was done maintaining the key dinucleotide composition and sequence direction using Nupack web resource. Resulting strands are presented in Table 1. Last, we added LNA to the resulting strands. LNAs were incorporated into the strands according to our previously reported design^19^, i.e. in a separated fashion along the entire strand, with one LNA in the middle and two near termini.

We constructed all possible dinucleotide repeats within a 21mer DNA, corrected them to avoid self-complementarity and hairpin formation and studied as double stranded complexes (Supplementary Table S5). GC rich sequences with GC% > 75% were rejected due to the formation of stable secondary structures. We also synthesized 10mer and 63mer oligonucleotides.

We initially tested five oligonucleotides as double-stranded helixes with different sequences^20^ (SEQ1–5; online Methods, Table 1). SEQ1, SEQ2 and SEQ3 are different in terms of nucleobase composition, and each contains three LNA modifications, at different positions. SEQ4 and SEQ5 have the same base sequences as SEQ1, but SEQ4 contains an additional LNA modification at nucleotide 10, which according to the original model, interacts with the lupus specific antibody ED-10, as does nucleotide 11. SEQ5 is an ordinary DNA.

**Figure 1:**
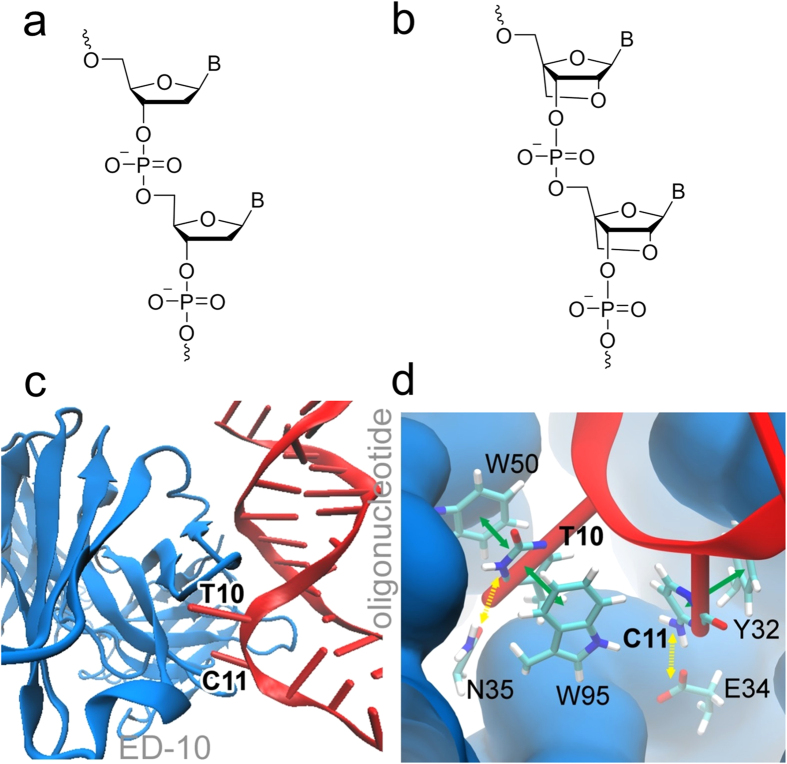
Chemical structures and key structural motifs for oligonucleotides and antibodies used in this study. Key motifs are shown for DNA (**a**) and LNA (**b**) oligomers; (**c**) Binding interactions between antibody ED-10 (blue) and oligonucleotide (red). (**d**) Binding of two nucleotides (here dTdC from SEQ1) in binding pocket of ED-10, showing π-stacking (solid green arrows) and hydrogen bonds (dashed yellow arrows).

We performed molecular simulations for SEQ1–5. The dsDNA (SEQ1-SEQ5) interacts with the antibody by unzipping the helix and sticking two nucleotides into the binding site of the antibody, as shown in Fig. 1c. As an example, a close-up view of the binding site of the antibody ED-10 in interaction with DNA (SEQ1) is shown in Fig. 1d. The strongest interactions suggested by the modelling involve the nucleotide thymine that forms a π-stacking motif with W50 and W95 from the antibody, followed by hydrogen-bonding interaction with N35. The second residue, which, depending on the DNA sequence SEQ1–5, is either a cytosine, adenine or guanine, interacts with the antibody in the binding site, and facilitates binding of the double helix through π-stacking with Y32 and the hydrogen-bonding interactions with E34 and G98. Hydrogen-bonding interactions with G91 and S99 are also possible in some cases. It is likely, however, that the π-stacking interactions between the nucleotides and the W50, W95 and Y32 residues provide more stabilisation of the DNA-antibody complex than the hydrogen-bonding interactions in the complex, because not all the hydrogen bonds were stable throughout the 100 ns simulation performed. The antibody’s hydrogen-bonding interactions with N35 and G98 are stable in all five oligonucleotides, but the stability of hydrogen bonds with E34, G91 and S99 depends on the kind of nucleobases of the two interacting nucleotides and whether the nucleotides are locked or not.

**Figure 2:**
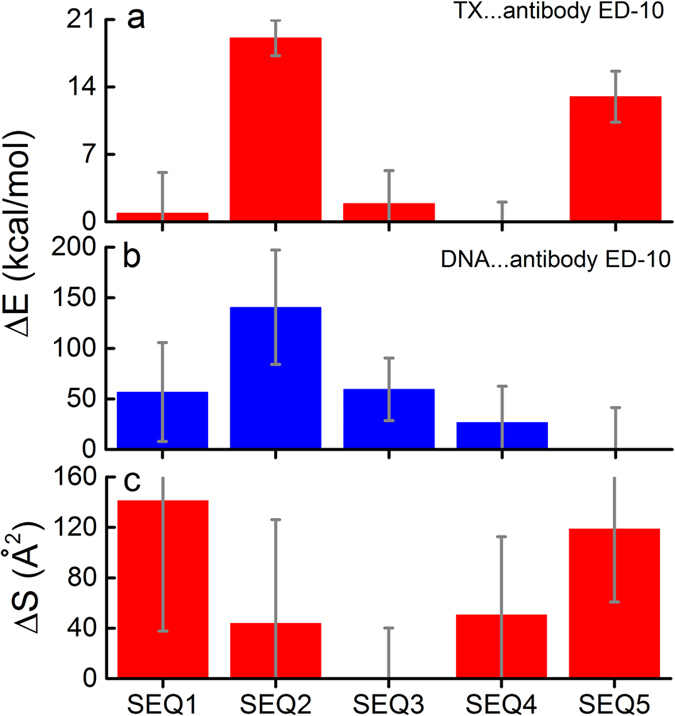
Bar charts showing the results of molecular modelling. (**a**) Bar charts showing the differences in the binding energy between the antibody ED-10 and residues 10 and 11 of oligonucleotides SEQ1-SEQ5, using the SEQ4 value as a reference point (−65.1 ± 2.1 kcal/mol). The symbol X in the legend is used to denote different bases which are either cytidine (SEQ1, SEQ4 and SEQ5), adenosine (SEQ2), guanosine (SEQ3). (**b**) Bar chart showing the differences in the binding energy between the antibody and the entire oligonucleotides (SEQ1-SEQ5), using the SEQ5 value as a reference point (−578.9 ± 83.0 kcal/mol). (**c**) Bar chart showing the differences in the contact area between the antibody ED-10 and the five oligonucleotides (SEQ1-SEQ5) using the SEQ3 value as a reference point (630.2 Å^2^). It should be noted that in all three graphs the standard deviation (SD) is used as error bars.

Next, the binding energy between the antibody ED-10 and the two nucleotides of the five oligonucleotides was calculated. Figure 2a shows this binding energy, and it turns out that the binding energy for SEQ4 is −65.1 ± 2.1 kcal/mol, the lowest energy value of the five sequences considered. SEQ1 and SEQ3 are also energetically similar, see Table 2, whereas SEQ2 and SEQ5 feature significantly higher energies (−46.1 ± 3.6 kcal/mol and −52.1 ± 5.2 kcal/mol, respectively), implying that the LNA modifications are lowering the binding energy in the binding site, except if the nucleobase is adenine, which does not fit properly into the binding site of the ED-10 antibody. It is also remarkable that the energy deviation of SEQ4 has the lowest value, indicating a more stable binding regime in this case. Both LNA modifications and the nucleobase composition are thus important for the specificity of DNA binding. However, the binding energies for SEQ1 and SEQ4 are very close, arguing that the extra LNA modification at thymine in position 10 of SEQ4 compared to SEQ1 only lowers the binding energy minimally. Thus an LNA modification at one of the nucleotides interacting with the antibody in the binding site does not seem to be essential for the binding energy.

The differences in the total binding energy between the antibody ED-10 and all dsDNA 21-mers were also calculated and analyzed (Fig. 2b). In this case, the total binding energy of the DNA with SEQ5 turns out to be the lowest (−578.9 ± 83.0 kcal/mol) and is, therefore, used as a reference point in the calculations presented in Fig. 2b. The result implies that the ordinary DNA binds better (has a higher association equilibrium constant to the antibody overall), but LNA modifications and some nucleobases optimise and lock the binding in the actual binding site, as also follows from Table 2. This locked binding is desirable, because it reduces cross-reactivity and stabilizes specific interactions, facilitating their detection. Ordinary DNA may bind best to ED-10 overall because it is flexible enough to bend around the antibody, thus creating more interactions with ED-10 than the oligonucleotides containing the LNA modifications. The LNA bases DNA-mimetics are more rigid and cannot bend in the same way as the ordinary DNA does^18^.

Another important characteristic to describe the binding affinity of the DNA and antibody is the contact area, S, which has been calculated, and is shown in Fig. 2c. The contact area is defined as the surface area, where the antibody and the oligonucleotide are in contact with each other. The antibody ED-10 has a surface area defined as S_protein_ and the surface area of the oligonucleotide is defined as S_nucleic acid_. When the antibody and the oligonucleotide interact with each other, they create a complex with a total surface area defined as S_protein + nucleic acid_. We are most interested in the surface area where the antibody and the oligonucleotide are in contact with each other. This surface area can be calculated by adding S_protein_ and S_nucleic acid_ followed by subtraction of S_protein + nucleic acid_, and can be computed as:


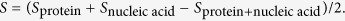


As expected, the calculations show that SEQ2, SEQ3 and SEQ4 have smaller contact areas between ED-10 and the oligonucleotide compared to the ordinary DNA, SEQ5 (see Table 2). SEQ1 has the biggest contact area with ED-10 (771.3 Å^2^), which is slightly larger than the contact area between ED-10 and SEQ5 (748.8 Å^2^). The deviation is, however, marginal, as the absolute values of the contact area can only be considered as qualitative measures. The analysis shows that LNA modifications lock the DNA/LNA molecules and could decrease the binding flexibility of the oligonucleotide. Thus, SEQ4, which displays a fairly low binding energy for the DNA-ED-10, does not have a particularly large contact area with ED-10. However, the contact area for the LNA containing SEQ1 is still high, suggesting that flexibility of oligonucleotide depends on the specific LNA modification. In addition, an LNA modification at one of the nucleotides interacting with the antibody in the binding site appears to be essential for the contact area, in contrast to the binding energy, where an LNA modification at that position did not seem to be essential.

The calculations provided a computational rationale for selecting antigen candidates for a novel diagnostic tool. The best candidate is defined as having the lowest binding energy upon binding with ED-10, especially upon binding with ED-10 in its particular binding site (Fig. 1d), as a sign of specificity, as well as having the largest contact area between the oligonucleotide and ED-10 (Fig. 2c). SEQ1 seems to be the best antigen candidate: it has nearly the lowest binding energy in the binding site, −64.2 kcal/mol, and a contact area value of 771.3 Å^2^ (see Table 2). SEQ5 (ordinary DNA) also seems to be promising, but the binding energy of this DNA to ED-10 is higher than the other oligonucleotides SEQ1, SEQ3 and SEQ4, being on average >10 kcal/mol higher. SEQ4 is the oligonucleotide with the lowest binding energy, being −65.2 kcal/mol, and is, therefore, also an interesting antigen candidate for further studies of antibodies to nucleic acids.

### SPR analysis of antibody binding to new antigens

We investigated the binding of synthetic DNA and LNA/DNA to autoantibodies using SPR. We immobilized biotin-modified double-stranded versions of SEQ4 and SEQ5 on a streptavidin coated SA chip and tested antibodies for binding. The rationale for choosing SEQ4 for the SPR study is that it contains an additional LNA modification that, according to the original model, is interacting with the lupus specific antibody ED-10 (Table 1). This allows us to study the effect of LNA positioned directly within the antibody binding site on the interaction and to compare it to the ordinary DNA of the same sequence, SEQ1. Monoclonal antibodies to dsDNA and β2-microglobulin were used as positive and negative controls, respectively. Polyclonal antibodies from 8 plasma samples obtained at Statens Serum Institute (SSI), Denmark, and negative control normal human plasma (HNP) were investigated by SPR (see Supplementary Table S3 for details on the subjects). Finally, serial dilutions of ED-10 were used for kinetic SPR assays^21^.

**Figure 3:**
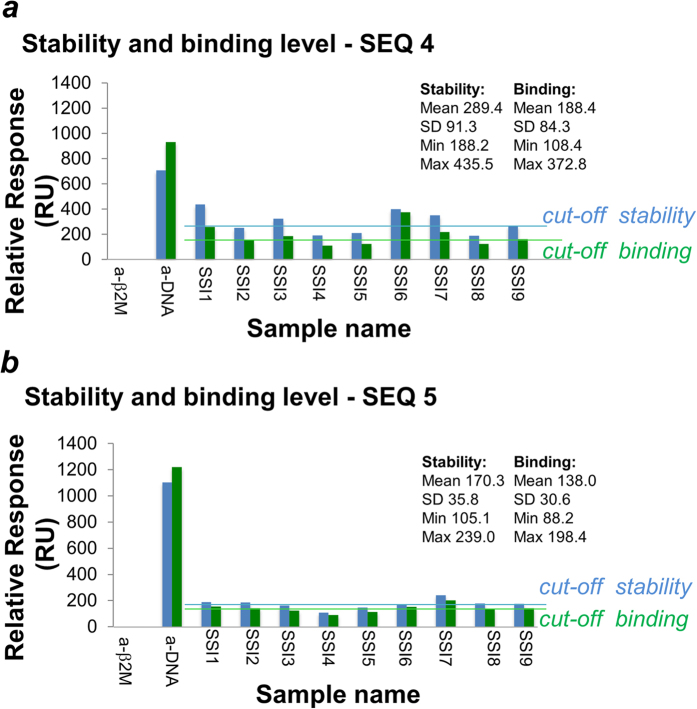
Stability and binding levels determined by SPR. Stability (blue bars) and binding (green bars) levels of autoantibodies in SLE sera were determined using SEQ4 (**a**) and SEQ5 (**b**). The levels were obtained by SPR assay in triplicates, using a chip modified with the stated antigens and sera in dilution 1:100. HNP (human normal plasma) is used as cut-off value. Descriptive statistics values (mean, SD; minimum and maximum values) and cut off are given for the polyclonal samples (SSI, HNP; n = 9).

The results of the binding assay, showing both binding and stability levels, are shown in Fig. 3. The binding level is the relative response just before the end of the sample injection and the stability level is the relative response just after the end of the sample injection. The stability level is used to identify stable or unstable binders by comparison with the value for the binding level. If there is a large drop in the relative response from the binding level to the stability level, the binder is unstable. The kIU/ml (kilo international unit/ml) values for the plasma samples determined by SSI are given in the experimental section. The cut-off value for a positive signal is 55 kIU/ml. The control monoclonal antibodies to dsDNA and β2-microglobulin have the highest and lowest RU binding values, respectively, validating the assay using SEQ5 as antigen. The 8 plasma samples had binding values in the interval 172–404 RU. If the RU binding value for HNP is used as a cut-off value, the plasma samples SSI1, SSI3, SSI6 and SSI7 show elevated binding. The results are reproducible, and the values shown in Fig. 3 are an average of two measurements with deviation of <5%. Statistical analysis of the stability and binding levels in the polyclonal samples (SSI) was done using descriptive statistics and Student’s t-test in R^22^. T-test showed that the difference in the binding values is not significant for SEQ4 and SEQ5, with p value 0.12. However, the difference between the stability values for SEQ4 and SEQ5 is significant with p = 0.0045.

Overall, SPR result does not correlate with the kIU/ml values from SSI. However the standard assay used by SSI is an assay with recombinant plasmid DNA in native double-stranded form without consistent sequence specificity, and thus there is a big difference between the two assays^23^.

Using SEQ4 as antigen, the monoclonal antibodies again have the highest and lowest RU binding values, respectively (Fig. 3a). The 8 plasma samples obtained binding values in the interval 88–200 RU; the results are reproducible and the values used in Fig. 3 are an average of two measurements. If the RU binding value for HNP is used as the cut-off value, plasma samples SSI1, SSI2, SSI6 and SSI7 show elevated binding with 19.5 RU standard deviation for replicated measurements. Very similar results are obtained using either SEQ4 or SEQ5 as antigen, with the exception of binding by SSI3 and SSI2. However, the RU values with these samples are similar and both are close to the cut-off value.

Based on SPR results, lower antibody binding levels are obtained using SEQ4 as the antigen compared to SEQ5 (Fig. 3). However, the monoclonal anti-dsDNA shows higher RU binding values using SEQ5. This suggests that the LNA modifications improve the binding to specific antibodies. This could be the explanation for the lower RU binding values obtained by the plasma samples using SEQ4 as antigen. The results for SEQ4 do not correlate with the kIU/mL values obtained by SSI (listed in the online Methods). The possible reasons for this discrepancy are same as those discussed when using SEQ5.

We performed a kinetic SPR assay (Supplementary Table S4)^21^. The results were analyzed using a 1:1 binding model as described by Buhl *et al*.^21^ The k_a_ values in biological systems are typically between 1*10^3^ Ms^−1^ and 1*10^7^ Ms^−1^, whereas k_d_ values are typically between 1*10^−1^ s^−1^ and 10^−6^ s^−1^^21^. As shown on Table 2, the calculated binding constants for autoantibodies to DNA are within these intervals, except for higher k_a_ value showed by SEQ4. The k_d_ for SEQ5 is lower than the k_d_ for SEQ4 by a factor of 10^3^. This indicates that ED-10 binds and dissociates much faster from SEQ4 than from SEQ5. Nevertheless, the dissociation constant (K_D_ value), which is a quantitative measurement of antibody affinity, is 7-fold lower for SEQ5 than SEQ4. These kinetic results correlate with the results from the binding SPR assays and the results of our simulations, which predict higher binding of LNA containing SEQ4 than for unmodified SEQ5 (Fig. 3a,b).

### ELISA confirms differences in the reactivity among DNA and LNA/DNA antigens

**Figure 4:**
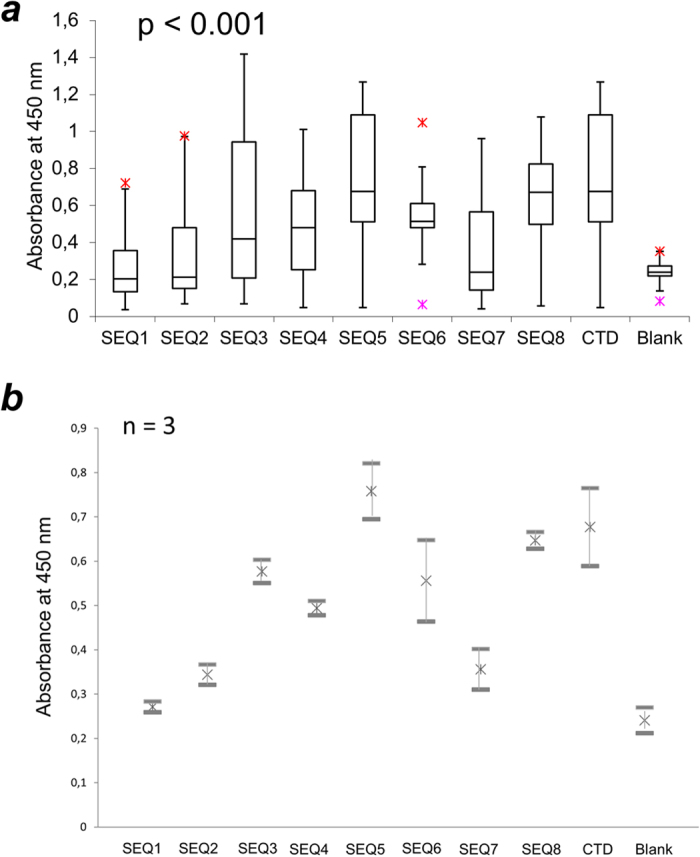
Results of ELISA assay for pediatric SLE subjects (n = 30), using DNA and LNA/DNA antigens. SEQ5–7 are pure DNA, whereas SEQ1–4 are their LNA/DNA analogues. In addition to SEQ1–5 described above, two LNA-free strands and G-quadruplex forming reference were used: 5′-ATT TAT TTT TAT ATT TAT ATT-3′, SEQ6; 5′-TGA ACT CTA TGT CTG TAT CAT-3′, SEQ7; 5′-TTAGGGTTAGGGTTAGGGTTAGGGTTAG-3′, SEQ8. CTD = calf thymus DNA. (**a**) Box-and-whisker plot with outliers. The arms on each boxplot are values Q_1_ − 1.5 × IQR and Q_3_ + 1.5 × IQR. Data points for each subject are means for three independent measurements. (**b**) Standard error bars for independent triplicate experiments (n = 3) across antigens; mean values for each data set are shown as well^22^,^24^.

Last, we aimed to develop an ELISA assay that takes advantage of rationally designed DNA and LNA/DNA antigens in detecting clinically relevant antibodies. Microtiter plates were coated with SEQ1–5, LNA-free duplexes of SEQ6–7 and control antigens (calf thymus DNA (CTD), and the G-quadruplex sequence used recently in the literature, SEQ8). We performed indirect ELISA with pediatric SLE plasma samples (n = 30) obtained from 27 subjects at Stanford University, USA (see Supplementary Table S3 for details on the subjects). Binding was observed as absorbance at 450 nm for TMB, a substrate oxidised by HPR enzyme (Fig. 4 and Supplementary Fig. S1). According to initial experiments, binding of antibodies by all antigens reached equilibrium in 1.5 h incubation at 37 °C (Supplementary Fig. S2). A linear decrease of signal was obtained in sample dilution 1:100−1:500 (Supplementary Fig. S3). Under these conditions, no background signal was observed for the empty plate incubated with SLE samples.

The ELISA results show that the effect of LNA modification on antibody binding is dependent on oligonucleotide sequence. Binding levels thus fall down upon LNA incorporation for SEQ1 vs. SEQ5 and SEQ2 vs. SEQ6. Conversely, the binding signal increases for SEQ3 vs. SEQ7. For SEQ4, the median signal is a higher signal for fewer samples and a smaller error bar compared to SEQ1. The overall difference between absorbance values for all antigens is statistically significant with p < 0.001 as determined by one-way ANOVA analysis. Importantly, the same samples show diverse reactivities with different constructs including CTD. When comparing antibody levels across antigens, SEQ4-SEQ6, SEQ8 and CTD have highest median values for binding. The difference in binding for SEQ1 and SEQ4 is statistically significant according to unpaired Student’s t-test (p < 0.05). In turn, the lowest signals are shown by AT-rich SEQ7 and LNA containing SEQ1–2. Notably, the results of LNA/DNA ELISA differ from those obtained by a clinical laboratory using Crithidia assay (p > 0.05 by ordinary least squares OLS test in R). Error bars are also large for LNA-free SEQ6 and CTD.

Another interesting observation is that for individual subjects the signal range becomes greater when LNA is introduced into the antigens. We studied this using data dispersity analysis (Supplementary Fig. S4). We collected absorbance values for each patient using antigens SEQ1–8 and CTD. Data dispersity was then calculated as the difference between the highest and lowest absorbance values of each sample^22^,^25^. As can be seen, data dispersity varies significantly for LNA modified antigens (Supplementary Fig. S4A), whereas non-modified sequences and controls show similar values (Supplementary Fig. S4B). Dispersity data was also subjected to descriptive statistical analysis in R. Thus, LNA resulted in standard deviation (SD) within a data set of 0.46 and mean value 0.44, whereas ordinary DNA showed smaller SD = 0.24 with a higher mean value of 0.67.

To further corroborate the value of our molecular design parameters, we prepared and evaluated additional DNA and LNA/DNA antigens (Supplementary Tables S5–S7). The variables tested were: nucleotide content, sequence direction, length and number/positions of LNA modifications. The sequences were tested for binding to monoclonal antibodies and randomly selected polyclonal samples (n = 6). Resulting data are shown in Supplementary Tables S8 and S9. We evaluated the interaction of antigens with the target based on discrimination of positive vs. negative SLE samples. Results of these experiments imply that SEQ1–5 have optimal discrimination. Increasing the length has only a minor effect on the binding, whereas shortening the antigen from 21 to 10 nucleotides results in a drop of binding. This is most likely due to the disturbance of the duplexes’ secondary structure. Sequence direction also had a high impact on the binding (see Supplementary Table S5, e.g. D1 compared to D5). We also observed a negative correlation of 5′-dTdC dinucleotide motif with the positive signal in the negative control, arguing that this motif indeed improves the specificity (Suppl. Table S11, positive correlation R (pos/neg)-n CT; p = 0.040, and a weak negative correlation M (neg) – n (CT) with p = 0.046).

Finally, we tested new antigens and controls using an expanded, unmatched healthy control cohort (n = 16; Supplemental Table S3). We observed increased signal for SEQ7–8 and CTD, with the highest median value for the binding by CTD (Supplementary Fig. S5). When compared to the rather low absorbance values observed for the synthetic antigens SEQ1–6, the difference was statistically significant with p < 0.001. This result additionally confirms the disease association of the positive signal for synthetic double-stranded antigens as compared to the previously reported G-quadruplex sequence and CTD.

## Discussion

The purpose of this work is to investigate the possibility of using novel LNA/DNA antigens as new reagents, which may have the potential to discriminate among anti-DNA antibodies and study their biology. As the results using LNA antigens differ from currently applied natural DNA, more verification is needed to propose the former as a diagnostic tool. Nevertheless, difference in the reactivity among DNA and LNA/DNA antigens does not conflict with recent reports on the specific structural requirements for nucleotide recognition by anti-DNA^9^.

Our rationale for the design of new antigens took into account several factors: sequence content, sequence polarity, length, presence and position of LNA modifications. Based on previous findings by us and others^10^,^19^, we built and tested three 21mers that contained zero-four LNA in the central and/or terminal positions. This work resulted in two major findings. First, interaction between antibodies and nucleic acids is strongly influenced by the sequence and chemical modification of nucleic acids. This finding concurs with previous data on chemically modified DNA; RNA and LNA aptamers, however this knowledge has not yet been applied to testing of anti-DNAs^26^,^27^,^28^,^29^,^30^. Second, advanced molecular modelling provides the necessary data on nucleic acid−antibody interaction that leads to biologically and potentially clinically valuable oligonucleotide antigens. So far computational studies of antibody−antigen binding have been done separately from biomedical assays. Our interdisciplinary approach simultaneously yields fundamental and practical information on nucleic acid antigens and in that way should be of benefit to both fields.

Molecular modelling revealed that the antibody ED-10 binds more strongly to specific sequences. SEQ1 (5′-TCC + TCT CTT **TC**T + CTT TCT + CTT-3′) obtained the best results in modelling, having both a large contact area (771.3 Å^2^) and a low binding energy at the binding site (−64.2 kcal/mol ± 8.4 kcal/mol) compared to the other four oligonucleotides. However, of all the five oligonucleotides, SEQ4 (5′-TCC + TCT CTT** + TC**T + CTT TCT + CTT-3′) achieved the lowest binding energy in the actual binding site (−65.1 kcal/mol ± 4.1 kcal/mol). This is a sign of highest specificity, which could lower the risk of cross-reactivity. Of note, SEQ4 and SEQ1 differ only by one extra LNA in the former.

To further corroborate the value of our molecular design parameters, we tested a series of antigens with a different sequence composition, sequence direction, length and LNA content. Our ELISA results for these sequences confirmed highest discrimination of the positive SLE samples vs. negative controls for SEQ1-SEQ5. We also observed that the binding was highly sensitive to these variables. Linear regression analysis additionally confirmed that a presence of 5′-dTdC-3′ dinucleotide motif within the antigen improves the correlation of the ELISA result with clinical manifestations of SLE. Importantly, even the duplex with similar nucleotide composition but different sequence directions had lower binding to the positive control antibody than the original design.

dTdC rich sequences without LNA show the highest number of positive results in SPR and ELISA studies. This correlates nicely with molecular modelling; however our SPR and ELISA profiles are rather different compared to those obtained by clinical laboratories and confirm altered antibody reactivity when the sequence of the antigen is changed. In addition to the sequence effect, the assays are performed at different temperatures: SSI is at 25 °C, whereas the SPR measurements are made at 37 °C. Using 37 °C better reflects physiologic interaction conditions and if some antibodies bind at 25 °C, but not at 37 °C, using 25 °C for the assays could give rise to false positive results. Overall, more analyses of patients samples together with clinical information are needed to conclude whether the results from the SPR and ELISA assays reported herein detect antibodies of a higher clinical relevance than current clinical diagnostics.

Another question is whether monoclonal antibodies are a true representation of the antibodies found in clinical samples. The selection of these antibodies may lead to a population of monoclonal antibodies that generally have a lower avidity towards DNA than is found for anti-DNA in sera^31^. Moreover, the constants are calculated using a 1:1 binding model, and it is debatable whether one can use this approximation, because antibodies have two binding sites^32^. Nevertheless, in contrast to bivalent models, data fitting with the 1:1 binding model allowed analysis of the interaction between synthetic dsDNA and autoantibodies and therefore has been adopted in this work^21^.

In conclusion, the results of both binding and kinetic assays indicate that the LNA modifications in short oligonucleotides increase the specificity and stability of the binding to antibodies related to SLE. Our study also confirms dTdC-dinucleotide preference for binding of DNA by autoimmune antibodies related to SLE. Although extended studies of human samples are needed to fully confirm specificity of the targeted antibodies to the disease, our method shows promise for increasing specificity of antibody detection^26^,^27^. In the future it would be exciting to expand the application of rationally designed LNA antigens to other targets, such as autoimmune antibodies in systemic sclerosis, rheumatoid arthritis and types of cancer that trigger anti-DNA immune responses^28^,^29^,^30^.

## Methods

### General

Biotin-modified oligonucleotides were purchased from Exiqon A/S, Vedbæk, Denmark. Oligonucleotides SEQ1–5 (Table 1) were synthesised in house using the phosphoramidite method^16^,^17^,^18^,^19^. All other nucleic acid compounds were obtained from Integrated DNA technologies, Inc., Iowa, USA. Monoclonal antibody controls (HYB 290-03 Anti-β2-Microglobulin (human) clone 12B2 and HYB 331-01 Anti double stranded DNA clone 35I9 available from BioPorto Diagnostics (Hellerup, Denmark) and human plasma samples from SLE subjects were from Statens Serum Institute (SSI), Copenhagen. Other samples were obtained as sera from Stanford University Hospital, California, USA (SLE subjects and unmatched healthy controls). Monoclonal antibody ED-10 was provided by Dr G. de Prat-Gay, Leloir Institute, Buenos Aires, Argentina.

SPR biosensor measurements were taken using Biacore X100. Chemicals used for biosensor measurements were purchased from GE Healthcare Life Sciences, Brøndby, Denmark. All other commercial reagents, buffers and solvents were purchased from Sigma-Aldrich Denmark ApS, Brøndby, Denmark. ELISA assays were made using a BRIO 2 ELISA processor at the Department of Clinical Biochemistry and Pharmacology, Odense University Hospital. 96-well Maxisorb NUNC microplates were purchased from Thermofisher Scientific.

### Modelling

To design effective oligonucleotide antigens, we performed a computational study of interactions between the five different 21-mer dsDNAs, both with and without LNA modifications, and the monoclonal antibody (ED-10) using the classical molecular dynamics (MD) approach. Sequences SEQ1–5 used in the simulation are shown in Table 1. Complementary strands: (SEQ1) 3′-AGG + AGA GAA AGA + GAA AGA + GTT-5′, (SEQ2) 3′-TAA + ATA AAA + ATA TAA ATA + TAA-5′, (SEQ3) 3′-ACT TGA + GAT ACA + GAC ATA + GTA-5′, (SEQ4) 3′-AGG + AGA GAA + AGA + GAA AGA + GTT-5′, (SEQ5) 3′-AGG AGA GAA AGA GAA AGA GTT-5′. The two bases indicated in bold (Table 1) were used to dock the dsDNA 21-mer to the antibody. The crystal structure of the antibody ED-10 binding 5′-dTdC-3′ was extracted from the PDB file 2OK0. The dTdC base pair in a simulated system from the preliminary study was aligned structurally with the dTdC base pair from the crystal structure, and the remaining part of the dsDNA 21-mer was then reconstructed using molecular building tools at hand^12^,^13^. To obtain the above-listed five sequences, the nucleotides from the earlier structure^9^ were substituted by the desired nucleotides and an additional bridge between O2′ and C4′ was added to the nucleotides to be locked^18^.

MD simulations were performed using NAMD 2.9^13^ with the CHARM36 force field for nucleic acids and proteins with CMAP corrections and the TIP3P water model^33^,^34^,^35^. In all simulations a 50 mM solution of NaCl was added to neutralise the system. Periodic boundary conditions were adopted in all MD simulations and the particle-mesh Ewald (PME) summation method was employed for evaluating Coulomb forces^36^,^37^. The van der Waals (vdW) energy was calculated using a smooth cutoff of 12 Å. The integration time step was 2 fs; temperature was kept at 310 K by applying Langevin forces with a damping coefficient of 5.0 ps^−1^ to all atoms in the system, except hydrogens. This computational protocol is rather standard and has been earlier applied to various molecular systems^38^,^39^,^40^. Each simulated system was first energy-minimised and then heated to 310 K. After heating, the simulated systems were first equilibrated for 0.1 ns with harmonic restraints applied to the protein and the DNA backbone, see Table 3. A relatively short equilibration time was used since the simulated system was largely inherited from a previously simulated structure, where post-equilibration production simulations were carried out for over 150 ns. Some of the DNA backbone was next released, but the phosphorous groups in the LNA/DNA were still fixed and the protein was still harmonically restrained. The system was simulated for a further 0.5 ns, following a simulation of 2 ns with only harmonic constraints applied to the DNA with some added artificial bonds between the bases of the nucleotides to preserve the complementarity of the duplex, as also follows from Table 3. Finally, all atoms were allowed to move and a further 5 ns of simulations were performed under NPT ensemble conditions and using Nosé-Andersen Langevin piston pressure control, allowing the systems to acquire a constant volume at 1 atm pressure and density prior to a 100 ns long production run simulation. The simulation protocol of the nucleic acid-ED10 complex is summarized in Table 3.

After equilibration, a 100 ns production simulation was carried out in the NVT ensemble, which was used for analysis. Note that the root-mean square displacement (RMSD) calculated for all atoms of the antibody proteins showed that the equilibration performed was sufficient to ensure a stable antibody structure. The molecular mutations and structure analysis were performed with VMD^12^, comprising calculation of the binding energies and contact areas between the antibody and the DNA/LNA duplexes as well as investigation of hydrogen bonds between the antibody and the DNA/LNA duplexes in the binding site.

### SPR studies

#### Oligonucleotides

The sequences of the used oligonucleotides were as follows: 5′-biotin-TCC TCT CTT TCT CTT TCT CTT-3′, complementary strand: 3′-AGG AGA GAA AGA GAA AGA GAA-5′; 5′-biotin-TCC + TCT CTT + TCT + CTT TCT + CTT-3′, complementary strand: 3′-AGG AGA GAA AGA GAA AGA GAA-5′, where the pluses indicate LNA modifications. Before immobilisation the duplexes were annealed for 10 min at 92 °C and then left at 4 °C for at least 4 hours.

#### Antibodies

The following antibodies were used; monoclonal antibodies: anti-dsDNA, anti-β2-microglubulin, ED-10; Human sera: SSI1 (103 kUI/mL), SSI2 (>200 kUI/mL), SSI3 (<35 kUI/mL), SSI4 (>200 kUI/mL), SSI5 (147 kUI/mL), SSI6 (>200 kUI/mL), SSI7 (132 kUI/mL), SSI8 (<35 kUI/mL); Control: HNP (obtained from Immunovision).

#### Immobilisation of antigens

The biotin-modified oligonucleotide (ligand) could efficiently be coupled to a SA chip surface with a 1 min injection of 140 μL aliquot at a flowrate of 30 μL/min using HBS-EP + (0.1 M HEPES, 1.5 M NaCl, 30 mM EDTA and 0.5% v/v Surfactant P20) as running buffer and the temperature kept at 37 °C. This step resulted in amide bond formation between streptavidin and biotin. After ligand injection a solution of 50% isopropanol in 50 mM NaOH and 1 M NaCl was injected. The surface was conditioned with 3 consecutive 1 minute injections of 1 M NaCl in 50 mM NaOH before the immobilisation. The oligonucleotide was only immobilised on Flow Cell (FC) 2. FC1 was used as a reference surface.

#### Biosensor measurements

The signal differences between the two flow cells were monitored as a function of time (as SPR sensorgrams), expressed in arbitrary resonance units (RU). One RU represents a change of 0.0001° in the angle of intensity minimum. For most proteins, including antibodies this is roughly equivalent to a change in concentration of about 1 pg/mm^2^ on the sensor surface. For the measurements of human sera and monoclonal antibody controls HBS-EP + (0.1 M HEPES, 1.5 M NaCl, 30 mM EDTA and 0.5% v/v Surfactant P20) was applied as a running buffer maintaining a continuous flow of 30 μL/min, and the temperature was kept at 37 °C. In binding assays, human sera were diluted 1:100 and monoclonal antibodies to a concentration of 100 mkg/mL with the respective running buffer prior to injection. 50 μL of the pre-diluted sera and monoclonal antibodies were conducted over the surface to allow association for 60 s, followed by another 60 s period of buffer injection to monitor dissociation of the immune complexes. In kinetic assays, antibody ED-10 was diluted to five different concentrations (2.5 mg/ml, 1.25 mg/ml, 0.63 mg/ml, 0.31 mg/ml and 0.16 mg/ml) with the respective running buffer prior to injection. Ninety μL of the pre-diluted antibodies were injected several times with a new concentration every time. The association was allowed for 60 s, followed by another 60 s period of buffer injection to monitor dissociation of the immune complex. Thereafter, in both binding and kinetic assay, the biosensor surface was regenerated with 30 s pulses of 50 mM NaOH/1 M NaCl until the signal returned to the baseline. The total measuring time for one serum/antibody sample was approx. 9 min. All buffers for the biosensor measurements were sterile-filtered using 0.2 micron FP CA-S filters (Whatman).

Statistical analysis of SPR data for polyclonal samples (SSI series and HNP control) was performed in R. This was done independently for the binding and stability levels using the descriptive statistics and two-sample Student’s t-test assuming unequal variances^22^.

### ELISA

In addition to SEQ1–5 described above, two LNA-free strands and G-quadruplex were used: 5′-ATT TAT TTT TAT ATT TAT ATT-3′, SEQ6; 5′-TGA ACT CTA TGT CTG TAT CAT-3′, SEQ7; 5′-TTAGGGTTAGGGTTAGGGTTAGGGTTAG-3′, SEQ8. The coating of 96 well ELISA plates was performed overnight using 2 μg/mL solution of a corresponding protein or conjugate in 1 × PBS (100 μL per well). After washing twice with 1 × PBS (300 μL), the plates were blocked for 1 h at RT with PTB buffer (50 μL Tween-20, 4 g BSA per 200 mL 1 × PBS; 100 μL per well). Plates were washed twice with 1 × PBS (300 μL) and incubated with plasma for 1.5 h at RT (100 μL per well). Plasma was diluted 1:100 using freshly prepared diluent (1 g BSA, 200 μL Tween-20 in 1 L 1 × PBS). After washing 3 times with 1 × PBS (300 μL) the second incubation was performed with a corresponding HRP-antibody (1.5 h, rt, 100 μL per well). The HRP-conjugate (a-IgG) was diluted 1:20.000 in the same diluent as for the initial incubation. Plates were washed 3 times with 1 × PBS (300 μL) and incubated with fresh TMB solution (100 μL/well) in 0.1 M acetate buffer (pH 5.4) for 15 min (100 μL/well). In doing this, 3 mg TMB was dissolved in 5 mL dimethyl sulfoxide (DMSO) and then diluted to 50 mL with 0.1 M acetate buffer containing 3 μL conc. H_2_O_2_. The reaction was stopped with 1 M H_2_SO_4_ (50 μL/well). Plates were analyzed using a Magellan TECAN microplate reader and measuring absorbance at 450 nm^41^.

Statistical analyses of ELISA data were performed in R using the following tests: one-way ANOVA of all absorbance values, unpaired Student’s t-test, ordinary least squares (OLS) for ELISA results vs. the data obtained by Crithidia assay; box-and-whisker plot analysis and error estimation; dispersity analysis^22^,^24^,^25^. The two-sample Student’s t-test was done assuming unequal variances^22^.

## Additional Information

**How to cite this article**: Samuelsen, S. V. *et al*. Synthetic oligonucleotide antigens modified with locked nucleic acids detect disease specific antibodies. *Sci. Rep*. **6**, 35827; doi: 10.1038/srep35827 (2016).

## Supplementary Material

Supplementary Information

## Figures and Tables

**Table 1:** Characteristics of the oligonucleotides used in simulations.

Sequence, 5′-3′	nr./position (LNAs)	Length (nt)	Base content
Sequences used in molecular modelling			
**SEQ1**: TCC + TCT CTT **TC**T + CTT TCT + CTT	3/4, 13, 19	21	62% T, 38% C
**SEQ2**: ATT + TAT TTT **+ TA**T ATT TAT + ATT	3/4, 10, 19	21	71% T, 29% A
**SEQ3**: TGA ACT + CTA **TG**T + CTG TAT + CAT	3/7, 13, 19	21	43% T, 24% A 19% C, 14% G
**SEQ4**: TCC + TCT CTT **+ TC**T + CTT TCT + CTT	4/4, 10, 14, 19	21	62% T, 38% C
**SEQ5**: TCC TCT CTT **TC**T CTT TCT CTT	0/−	21	62% T, 38% C

The five oligonucleotides have different sequences and additional LNA in SEQ1-SEQ4*.

*Pluses indicate LNA modifications. Bold indicate docking bases.

**Table 2:** Total binding energies and contact area values obtained for SEQ1-SEQ5, and shown in Fig. 2.

Sequence	E TX … Protein (kcal/mol)	E DNA … Protein (kcal/mol)	S (Å^2^)	n
SEQ1	−64.2 ± 8.5	−522.1 ± 97.5	771.3 ± 103.4	2001
SEQ2	−46.1 ± 3.7	−438.3 ± 112.9	674.1 ± 82.2	2001
SEQ3	−63.2 ± 6.9	−519.4 ± 61.8	630.2 ± 40.2	786
SEQ4	−65.1 ± 4.1	−552.1 ± 71.7	680-7 ± 62.1	1774
SEQ5	−52.1 ± 5.3	−578.9 ± 82.9	748.8 ± 57.9	1812

Note that the values in Fig. 2 are plotted relative to the smallest value of energy or the contact area. Additionally, the number of points (n) used in the averaging for SEQ1-SEQ5 is indicated.

**Table 3:** Protocol for nucleic acid‒target simulations.

Process	Time interval (ns)
Structure minimization	2500 NAMD steps
DNA side chains, water molecules and ions released; rest fixed	0.1
DNA backbone except phosphorous atoms released but with some artificial bonds between complementary nucleobases; protein harmonic restrained	0.5
Harmonic constraints applied to DNA with some artificial bonds between complementary nucleobases	2
All atoms released	5
NVT ensemble, production simulation	100

The simulations were carried out in the present study for each of the DNA and LNA/DNA sequences summarized in Table 1 and antibody ED-10.
