# Supplementary material for: Synthetic oligonucleotide antigens modified with locked nucleic acids detect disease specific antibodies
Source: Sci Rep. 2016 Oct 24;6:35827. doi: 10.1038/srep35827 (PMC5075775; doi:10.1038/srep35827)
Supplement: Supplementary Information [file srep35827-s1.doc]

**Supplementary Information for the paper:**

**Synthetic oligonucleotide antigens modified with locked nucleic acids detect disease specific antibodies**

Simone V. Samuelsen, Ilia A. Solov’yov*, Imelda M. Balboni, Elizabeth Mellins, Christoffer Tandrup Nielsen, Niels H. H. Heegaard and Kira Astakhova*

**List of abbreviations**

DNA Deoxyribonucleic acid

Ig Immunoglobulin

SLE Systemic lupus erythematosus

ss Single-stranded

ds Double-stranded

Arg Arginine

D-ARM ssDNA-antibody recognition module

ANA Antinuclear antibodies

ELISA Enzyme-linked immunosorbent assay

IIF Indirect immunofluorescence

RIA Radioimmunoassay

LNA Locked nucleic acid

DMT 4,4’-Dimethoxytrityl

VMD Visual molecular dynamics
NAMD Nanoscale molecular dynamics program

Ab Antibody

mAb Monoclonal antibody

SPR Surface plasmon resonance

SA Streptavidin

HNP Human normal plasma

HSS Human control plasma, anti-ssDNA positive

HDD Human control plasma, anti-dsDNA positive

OUH Odense Universitetshospital

TMB 3,3’,5,5’-Tetramethylbenzidine

SSI Statens serum institut

RU Relative response unit

MD Molecular dynamics

RMSD Root-mean-square deviation

EDTA Ethylenediaminetetraacetic acid

HEPES 4-(2-hydroxyethyl)-1-piperazineethanesulfonic acid

FC Flow cell

PBS Phosphate-buffered saline

BSA Bovine serum albumin

HRP Horseradish peroxidase

DMSO Dimethyl sulfoxide

RT Room temperature

KUI Kilo international unit

Lys Lysine

His Histidine

Trp Tryptophan

Asn Asparagine

Tyr Tyrosine

Ser Serine

Thr Threonine

Gly Glycine

**Supplementary Table S1. Antigens cross-reacting with anti-DNA antibodies.*[[1]](#footnote-2)**

| Antigen | Cross-reactivity with anti-dsDNA | Cross-reactivity with anti-ssDNA |
| --- | --- | --- |
| Z-DNA | + | + |
| Cardiolipin | + | + |
| Phosphatidylglycerol | ND* | + |
| Bacterial phospholipids | + | ND |
| Pneumococcal phosphorylcholine | + | ND |
| Mycobacterial glycolipid | + | + |
| Klebsiella polysaccharides | + | + |
| Meningococcal polysaccharides | + | + |
| Pneumococcal polysaccharides | + | ND |
| E. Coli polysaccharides | + | + |
| Hyaluronic acid | + | ND |
| Chondroitin sulfate | + | ND |
| Heparan sulfate | + | ND |
| Laminin | + | + |
| Cell surface proteins | + | ND |
| Vimentin | + | + |
| Ribosomal proteins S1, P0, P1 | + | ND |
| Small nuclear ribonucleoprotein A and D | + | ND |

* ND: not defined

**Supplementary Table S2.** Distributions of interaction types in the interaction sites of protein-DNA complexes.[[2]](#footnote-3)[[3]](#footnote-4)

| **Interaction type** | **Distribution (%)** | **Energy (kJ/mol)** |
| --- | --- | --- |
| Hydrogen bonds | 51 | 20 (5-40) |
| Electrostatic | 8 | 20 (10-50) |
| Hydrophobic | 19 | <40 |
| Van der Waals | 22 | 1. (1-20) |

**Supplementary Table S3. Subjects clinical parameters.**

| **Variable** | **Subjects data (n = 52)** |
| --- | --- |
| Healthy controls | 16 |
| SU cohort | 27 |
| SSI cohort | 8 |
| SLE positive by clinical evaluation, SU and SSI cohort | 35 |
| dsDNA positive (CTD assay done in house) | 21 (60%) |
| dsDNA positive (Crithidia done in clinical lab) | 20 (57%) |
| Mean (range) SLEDAI score | 11.6 (4-30)* |
| Class III/IV lupus nephritis-biopsy proven† (%) | 10 (37)* |
| Mean time from disease onset till sample collection (range) | 7 months (0-36 months)* |

* Data available for SUH cohort, n = 27

**Supplementary Table S4. Results of kinetic SPR assay for ED-10 binding to SEQ4 and SEQ5.**

| **Value** | **SEQ4 (LNA/DNA)** | **SEQ5 (DNA)** |
| --- | --- | --- |
| **k_a_** | 1.009*10^8^ Ms^-1^ | 3.341*10^3^ Ms^-1^ |
| **k_d_** | 7.860*10^-1^ s^-1^ | 3.531*10^-4^ s^-1^ |
| **K_D_** | 7.787*10^-7^ M | 1.057*10^-7^ M |

**Supplementary Table S5.** Oligonucleotide design and ELISA results for the dsDNA sequences of diverse dinucleotide motif composition.*

| De-sign # | Dinuc-leotide motif,  5’-3’ | Sequence1, 5’-3’** | Absorbance at 450 nm | | | | | | |
| --- | --- | --- | --- | --- | --- | --- | --- | --- | --- |
|  |  |  | HNP | HC1 | HC2 | Anti-dsDNA | HDD | SSI6 | SSI7 |
| dsDNA antigens with dinucleotide pair 1[[4]](#footnote-5) | | |  |  |  |  |  |  |  |
| D1^§^ | TC | TCC TCT CTT TCT CTT TCT CTT | 0.22 | 0.31 | 0.24 | 1.41 | 0.54 | 0.89 | 1.21 |
| D2 | TA | TAA TAT ATT TAT ATT TAT ATT | 0.37 | 0.65 | 0.23 | 1.11 | 0.43 | 1.05 | 0.64 |
| D3 | TG | TGG TGT GTT TGT GTT TGT GTT | 0.40 | 0.76 | 0.32 | 1.13 | 0.56 | 1.05 | 1.17 |
| D4 | TT | TTT TTT TTT TTT TTT TTT TTT | 0.45 | 0.27 | 0.50 | 0.66 | 0.67 | 0.80 | 0.77 |
| dsDNA antigens with reversed sequence direction to SEQ no. 1-3 | | |  |  |  |  |  |  |  |
| D5 | CT | TTC TCT TTC TCT TTC TCT CCT | 0.20 | 0.18 | 0.32 | 0.98 | 0.19 | 0.77 | 0.97 |
| D6 | AT | TTA TAT TTA TAT TTA TAT AAT | 0.31 | 0.75 | 0.28 | 1.00 | 0.55 | 0.85 | 0.64 |
| D7 | GT | TTG TGT TTG TGT TTG TGT GGT | 0.70 | 0.56 | 0.54 | 1.87 | 0.89 | 1.11 | 1.27 |
| dsDNA antigens with different dinucleotide pairs | | |  |  |  |  |  |  |  |
| D8^§^ | AT | ATT TAT TTT TAT ATT TAT ATT | 0.27 | 0.22 | 0.25 | 1.27 | 0.31 | 1.05 | 0.87 |
| D9 | AC | ACC CAC CCC CAC ACC CAC ACC | 0.55 | 0.37 | 0.48 | 0.67 | 0.65 | 0.70 | 1.03 |
| D10 | AA | AAA AAA AAA AAA AAA AAA AAA | 0.32 | 0.55 | 0.89 | 0.77 | 0.57 | 0.56 | 0.60 |
| D11 | AG | AGG GAG GGG GAG AGG GAG AGG | 0.54 | 0.30 | 0.22 | 0.78 | 0.56 | 0.88 | 1.01 |
| dsDNA with reversed sequence direction to SEQ no. 9,11 | | |  |  |  |  |  |  |  |
| D12 | CA | CCA CAC CCA CAC CCC CAC CCA | 0.24 | 0.17 | 0.29 | 0.78 | 0.54 | 1.00 | 0.99 |
| D13 | GA | GGA GAG GGA GAG GGG GAG GGA | 0.24 | 0.20 | 036 | 0.89 | 0.35 | 0.70 | 1.21 |
| ds mixmer control | | |  |  |  |  |  |  |  |
| D14^§^ | ATCG | TGA ACT CTA TGT CTG TAT CAT | 0.21 | 0.17 | 0.31 | 1.55 | 0.40 | 1.32 | 1.16 |

* The key dinucleotide was designed by the mutation of the strands shown in Table 1 followed by eliminating of self-complementarity and hairpin formation using Nupack. ELISA was performed for the resulting strands as duplexes with corresponding complementary DNA. Samples for this assay were selected randomly from healthy control and SSI SLE cohorts using R. For ELISA conditions, see methods.

** Complementary sequences for each duplex are not presented.

^§^ D1, D8 and D14 correspond to SEQ5,SEQ6 and SEQ7 in the paper, respectively (Table 1).

**Supplementary Table S6.** Oligonucleotide design and ELISA results for the dsDNA sequences of diverse length.

| De-sign # | Dinuc-leotide motif,  5’-3’ | Sequence 5’-3’ * | Absorbance at 450 nm* | | | | | | |
| --- | --- | --- | --- | --- | --- | --- | --- | --- | --- |
|  |  |  | HNP | HC1 | HC2 | Anti-dsDNA | HDD | SSI6 | SSI7 |
| 10mer dsDNA | | |  |  |  |  |  |  |  |
| D15 | TC | TCC TCT CTT T | 0.22 | 0.31 | 0.24 | 0.90 | 0.34 | 0.89 | 0.65 |
| D16 | AT | ATT TAT TTT T | 0.27 | 0.22 | 0.25 | 0.76 | 0.19 | 0.87 | 0.56 |
| 63mer dsDNA | | |  |  |  |  |  |  |  |
| D17 | TC | TCC TCT CTT TCT CTT TCT CTT TCC TCT CTT TCT CTT TCT CTT TCC TCT CTT TCT CTT TCT CTT | 0.26 | 0.19 | 0.27 | 1.50 | 0.45 | 1.01 | 1.54 |
| D18 | AT | ATT TAT TTT TAT ATT TAT ATT ATT TAT TTT TAT ATT TAT ATT ATT TAT TTT TAT ATT TAT ATT | 0.23 | 0.30 | 0.24 | 1.44 | 0.40 | 1.25 | 0.99 |

***** Complementary sequences for each duplex are not presented.

**Supplementary Table S7.** Oligonucleotide design and ELISA results for the dsDNA sequences containing LNA.*

| De-sign # | Dinuc-leotide motif,  5’-3’ | Sequence 5’-3’ ** | Absorbance at 450 nm* | | | | | | |
| --- | --- | --- | --- | --- | --- | --- | --- | --- | --- |
|  |  |  | HNP | HC1 | HC2 | Anti-dsDNA | HDD | SSI6 | SSI7 |
| ds LNA/DNA | | |  |  |  |  |  |  |  |
| D19^§^ | TC | TCC +TCT CTT TCT +CTT TCT +CTT | 0.22 | 0.16 | 0.20 | 1.12 | 0.19 | 0.32 | 0.95 |
| D20 | TC | +TCC +TCT CTT TCT CTT TCT CTT | 0.18 | 0.22 | 0.20 | 0.89 | 0.21 | 0.18 | 0.67 |
| D21 | TC | TCC TCT CTT T+CT +CTT TCT CTT | 0.20 | 0.37 | 0.20 | 0.77 | 0.54 | 0.19 | 0.78 |
| D22 | TC | TCC +TCT CTT TCT CTT TCT CTT | 0.22 | 0.30 | 0.17 | 0.56 | 0.32 | 0.25 | 0.54 |

* LNA nucleotides are marked with plus before the corresponding letter. ** Complementary sequences for each duplex are not presented.

^§^ D19 corresponds to SEQ1 in the paper (Table 1).

**Supplementary Table S8.** Data analysis of ELISA results for the double stranded antigens having different sequence content.*

| Design # | Dinucleotide motif, 5’-3’ | | M (pos) | M (neg) | R (pos/neg) |
| --- | --- | --- | --- | --- | --- |
| 1 | TC |  | 1.17 | 0.33 | 3.57 |
| 2 | TA |  | 0.93 | 0.42 | 2.22 |
| 3 | TG |  | 1.12 | 0.51 | 2.19 |
| 4 | TT |  | 0.74 | 0.47 | 1.57 |
| 5 | CT |  | 0.91 | 0.22 | 4.07 |
| 6 | AT |  | 0.83 | 0.47 | 1.76 |
| 7 | GT |  | 1.42 | 0.67 | 2.11 |
| 8 | AT |  | 1.06 | 0.26 | 4.05 |
| 9 | AC |  | 0.80 | 0.51 | 1.56 |
| 10 | AA |  | 0.64 | 0.58 | 1.10 |
| 11 | AG |  | 0.89 | 0.41 | 2.20 |
| 12 | CA |  | 0.92 | 0.31 | 2.98 |
| 13 | GA |  | 0.93 | 0.29 | 3.25 |
| 14 | ATCG |  | 1.34 | 0.27 | 4.93 |

***** M (pos) and M (neg) are mean values for the absorbance of SLE positive and negative samples; R (pos/neg) is a discrimination factor between the mean positive and mean negative values, i.e. R (pos/neg) = M(pos)/M(neg).

**Supplementary Table S9.** Data analysis of ELISa results for the double stranded antigens having different sequence length and LNA modifications.*

| Design # | Dinucleotide motif, 5’-3’ | | M (pos) | M (neg) | R (pos/neg) |  |
| --- | --- | --- | --- | --- | --- | --- |
| 15 | TC |  | 0.81 | 0.28 | 2.93 |  |
| 16 | AT |  | 0.73 | 0.23 | 3.14 |  |
| 17 | TC |  | 1.35 | 0.29 | 4.62 |  |
| 18 | AT |  | 1.23 | 0.29 | 4.19 |  |
| 19 | TC |  | 0.80 | 0.19 | 4.14 |  |
| 20 | TC |  | 0.58 | 0.20 | 2.86 |  |
| 21 | TC |  | 0.58 | 0.33 | 1.77 |  |
| 22 | TC |  | 0.45 | 0.25 | 1.78 |  |

***** M (pos) and M (neg) are mean values for the absorbance of SLE positive and negative samples; R (pos/neg) is a discrimination factor between the mean positive and mean negative values, i.e. R (pos/neg) = M(pos)/M(neg).

**Supplementary Table S10.** Input data for the linear regression analysis.*

| Design # | M (pos) | M (neg) | R (pos/neg) | T_m_, °C ** | n (CT) | n (TA) |
| --- | --- | --- | --- | --- | --- | --- |
| 1 | 1.17 | 0.33 | 3.57 | 51 | 7 | 0 |
| 2 | 0.93 | 0.42 | 2.22 | **34** | 0 | 7 |
| 3 | 1.12 | 0.51 | 2.19 | 53 | 0 | 0 |
| 4 | 0.74 | 0.47 | 1.57 | **39** | 0 | 0 |
| 5 | 0.91 | 0.22 | 4.07 | 50.5 | 7 | 0 |
| 6 | 0.83 | 0.47 | 1.76 | **34** | 0 | 7 |
| 7 | 1.42 | 0.67 | 2.11 | 52.5 | 0 | 0 |
| 8 | 1.06 | 0.26 | 4.05 | **34** | 0 | 5 |
| 9 | 0.8 | 0.51 | 1.56 | 66 | 0 | 0 |
| 10 | 0.64 | 0.58 | 1.1 | **39** | 0 | 0 |
| 11 | 0.89 | 0.41 | 2.2 | 65 | 0 | 0 |
| 12 | 0.92 | 0.31 | 2.98 | 66 | 0 | 0 |
| 13 | 0.93 | 0.29 | 3.25 | 65 | 0 | 0 |
| 14 | 1.34 | 0.27 | 4.93 | 46.5 | 3 | 1 |
| 15 | 0.81 | 0.28 | 2.93 | **24** | 3 | 0 |
| 16 | 0.73 | 0.23 | 3.14 | **8** | 0 | 1 |
| 17 | 1.35 | 0.29 | 4.62 | 80 | 21 | 0 |
| 18 | 1.23 | 0.29 | 4.19 | 61 | 0 | 21 |
| 19 | 0.8 | 0.19 | 4.14 | 63 | 7 | 0 |
| 20 | 0.58 | 0.2 | 2.86 | 58.5 | 7 | 0 |
| 21 | 0.58 | 0.33 | 1.77 | 56 | 7 | 0 |
| 22 | 0.45 | 0.25 | 1.78 | 52 | 7 | 0 |

*T_m_ = thermal denaturation value; T_m_ were determined in a medium salt phosphate buffer (pH 7.2), as described.[[5]](#footnote-6) M (pos) and M (neg) are mean values for the absorbance of SLE positive and negative samples; R (pos/neg) is a discrimination factor between the mean positive and mean negative values, i.e. R (pos/neg) = M(pos)/M(neg). n (CT/TA) = number of corresponding base pairs per antigen sequence.

** According to the T_m_ data, duplexes 2,4,6,8,10 melted near the temperature of the ELISA assay (37 °C), whereas the duplexes 15-16 were not formed at 37 °C. Nevertheless, the pre-annealed mixture of the two complementary strands was used in ELISA for these antigens.

**Supplementary Table S11. Results of the linear regression analysis.***

|  |  |  | Correlated variables: | | | | | | |
| --- | --- | --- | --- | --- | --- | --- | --- | --- | --- |
|  | M (pos) - T_m_ | M (neg) -T_m_ | R (pos/neg) - T_m_ | M (pos) – n (CT) | M (neg) - n (CT) | R (pos/neg) - n (CT) | M (pos) – n (TA) | M (neg) - n (TA) | R (pos/neg) - n (TA) |
| p | 0.280 | 0.899 | 0.413 | 0.662 | **0.046**^§^ | **0.040**^§^ | 0.247 | 0.808 | 0.345 |
| CI, 95% | 0.015 | 0.007 | 0.062 | 0.049 | 0.022 | 0.187 | 0.050 | 0.026 | 0.212 |

***** CI = confidence interval; n (CT/TA) = number of corresponding base pairs per antigen sequence. For the description of other variables, see Table S10.

^§^ For M (neg) – n (CT), a negative correlation was observed; for R (pos/neg) - n (CT), the correlation was positive.

**Supplementary Figure S1.** Main principle of currently applied a-DNA detection methods: ELISA (A), Crithidia luciliae (B) and RIA (C).

**a b**

**
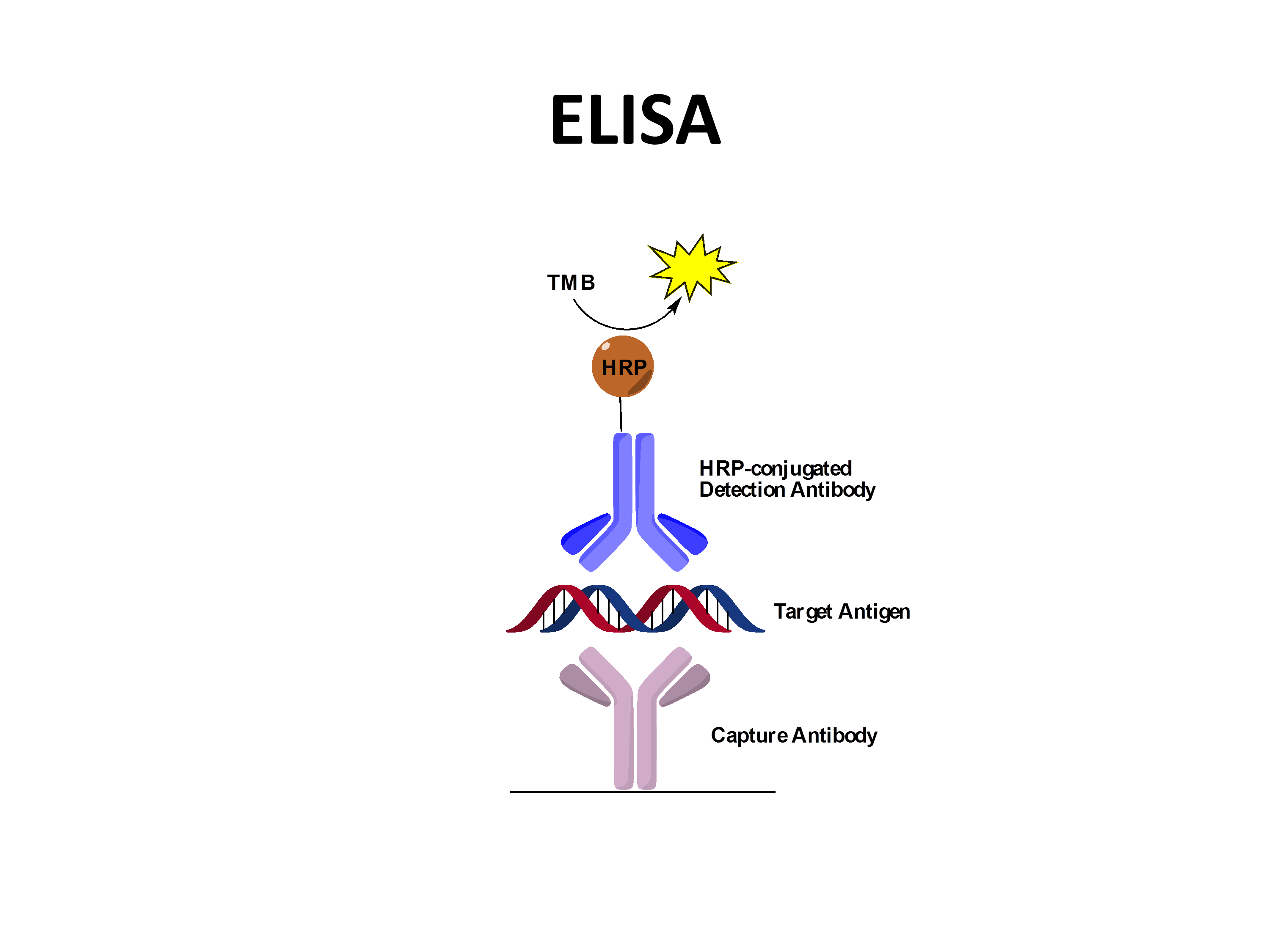

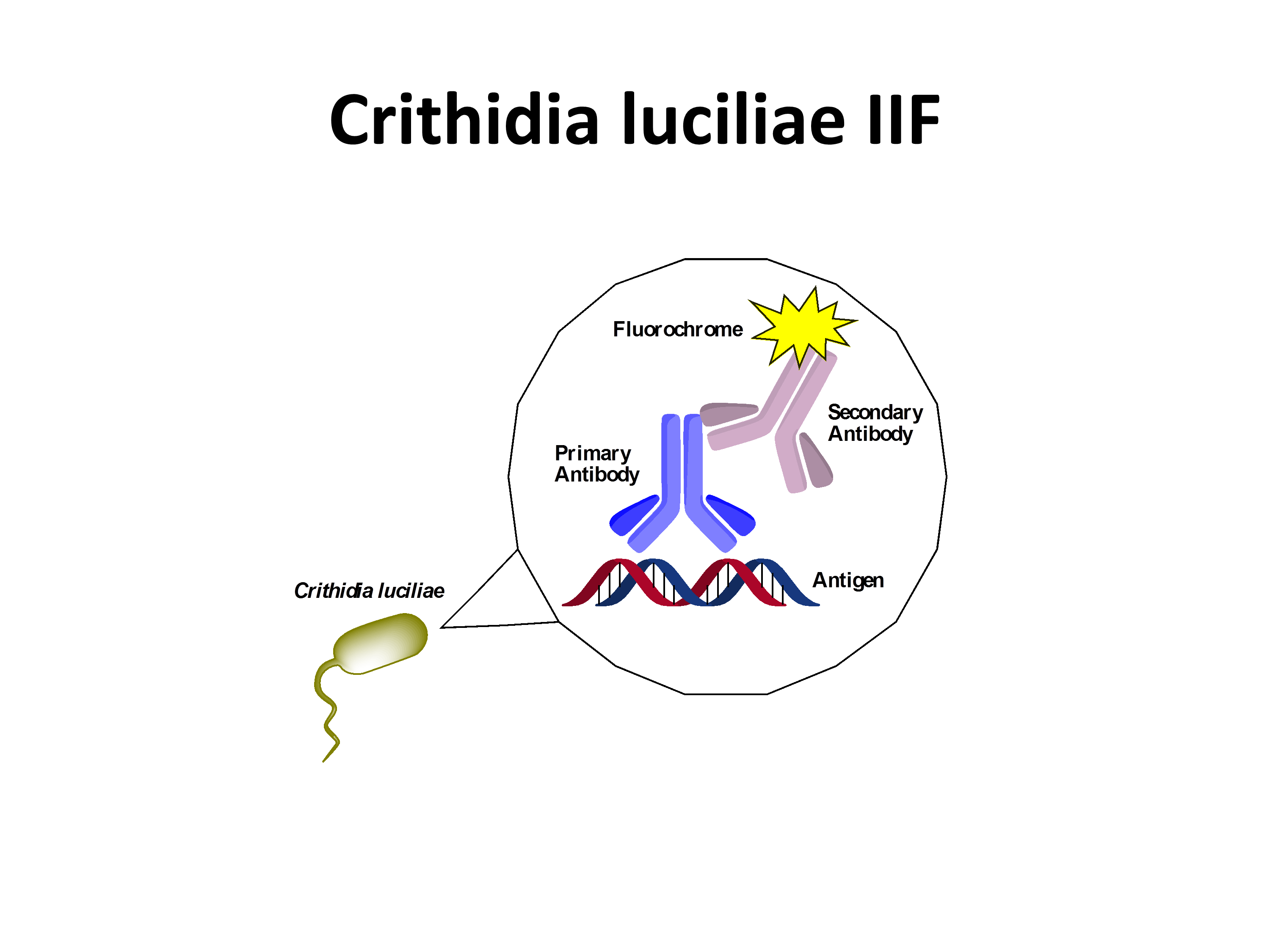
**

**c
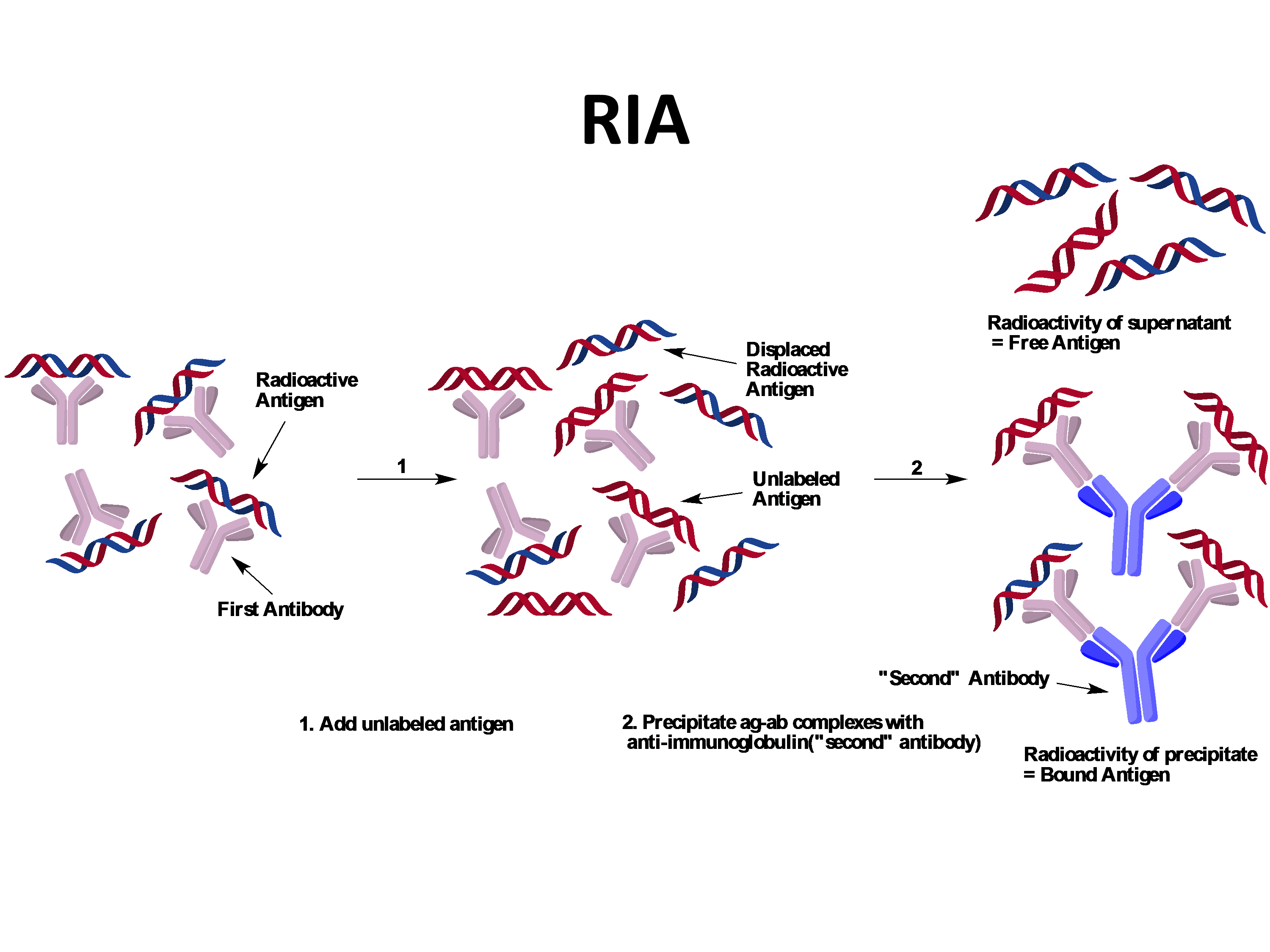
**

**Supplementary Figure S2.** Binding equilibrium time study for polyclonal controls and randomly selected patient plasma samples with antigens used in this study.*

**a** SEQ1


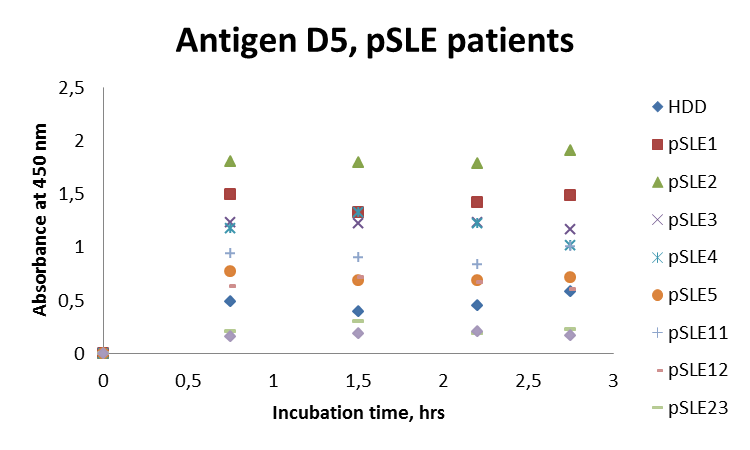


**b** CTD


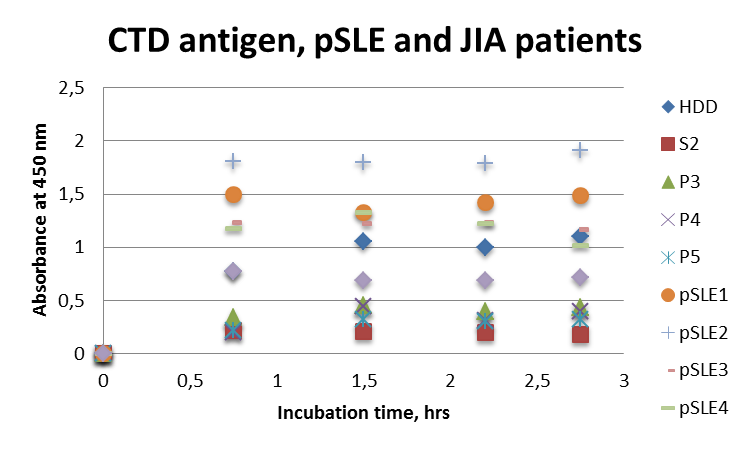


* P3, P4 and P5 are patient samples from SSI cohort.

**Supplementary Figure S3.** Plasma titration curves for polyclonal controls (IgG assay).*


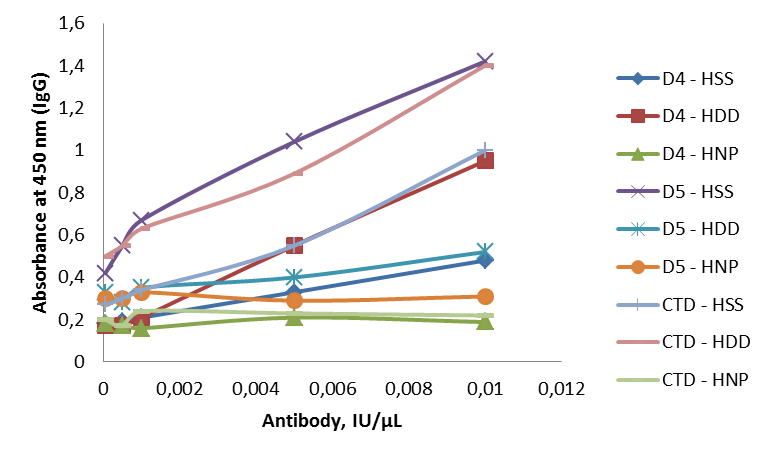


* D4 = DNA SEQ4; D5 = DNA SEQ5. For the sequences, see Table 1.

**Supplementary Figure S4.** Data dispersity analysis for LNA/DNA (A) and DNA (B) antigens. Data points were obtained by ELISA assay in sera dilution 1:100, SU cohort.

**a**


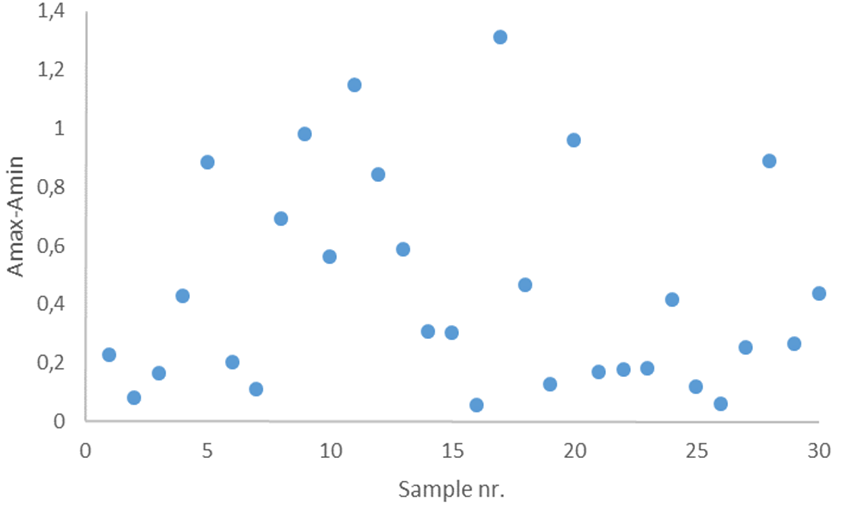


**b**


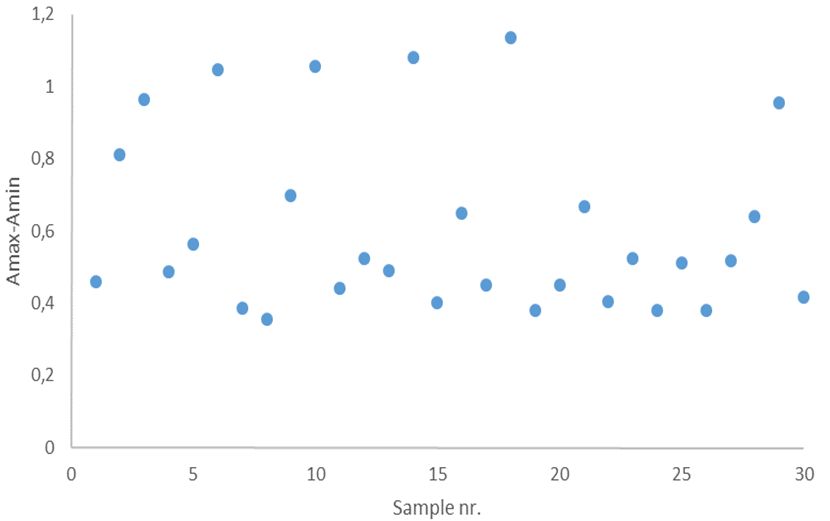


Supplemental Figure S5. ELISA results for extended HC cohort (n = 16). A) box-and-whisker plot of absorbance values for each antigen with outliers; B) Mean values and standard error bars for the three independent measurements.


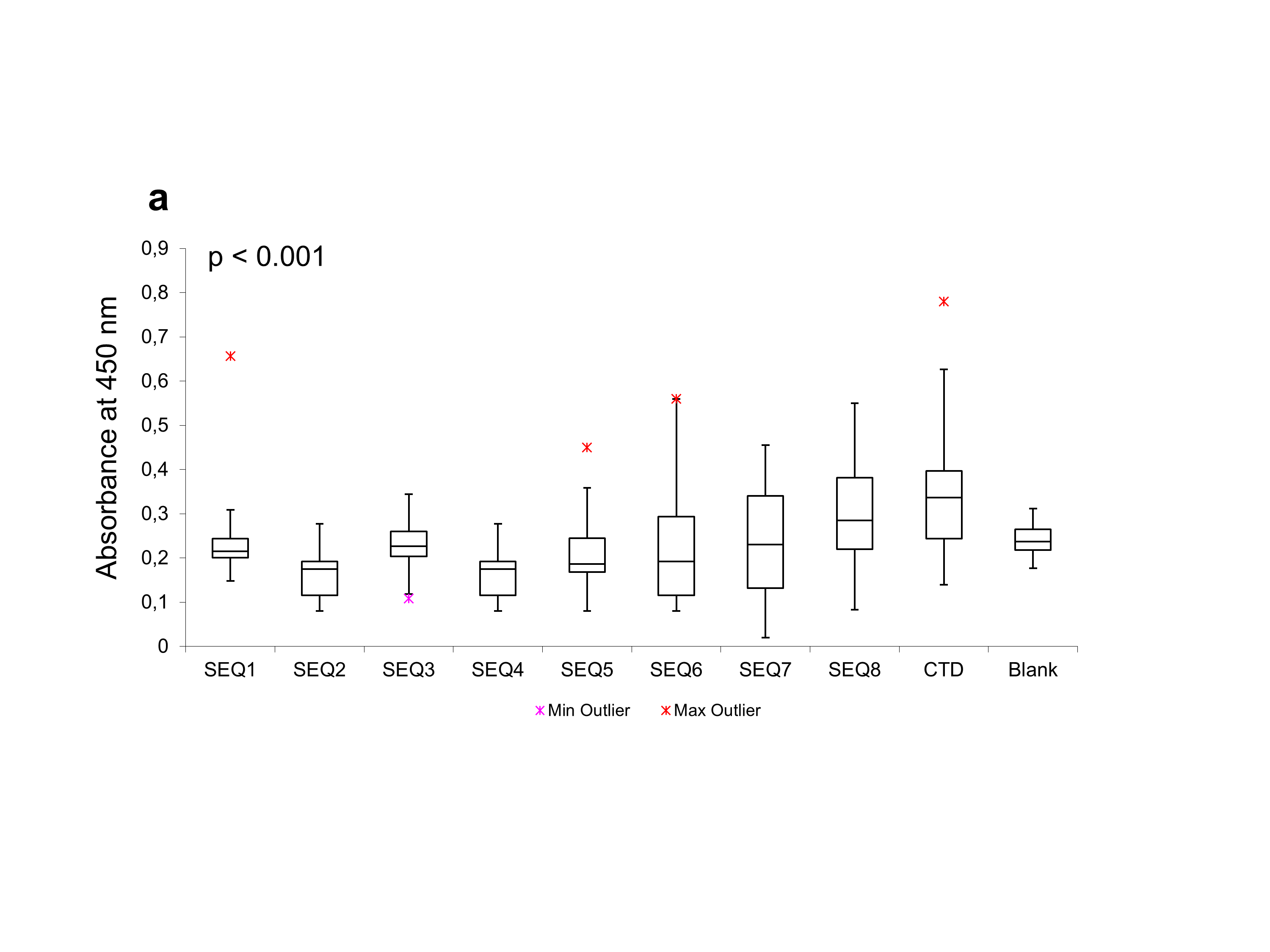


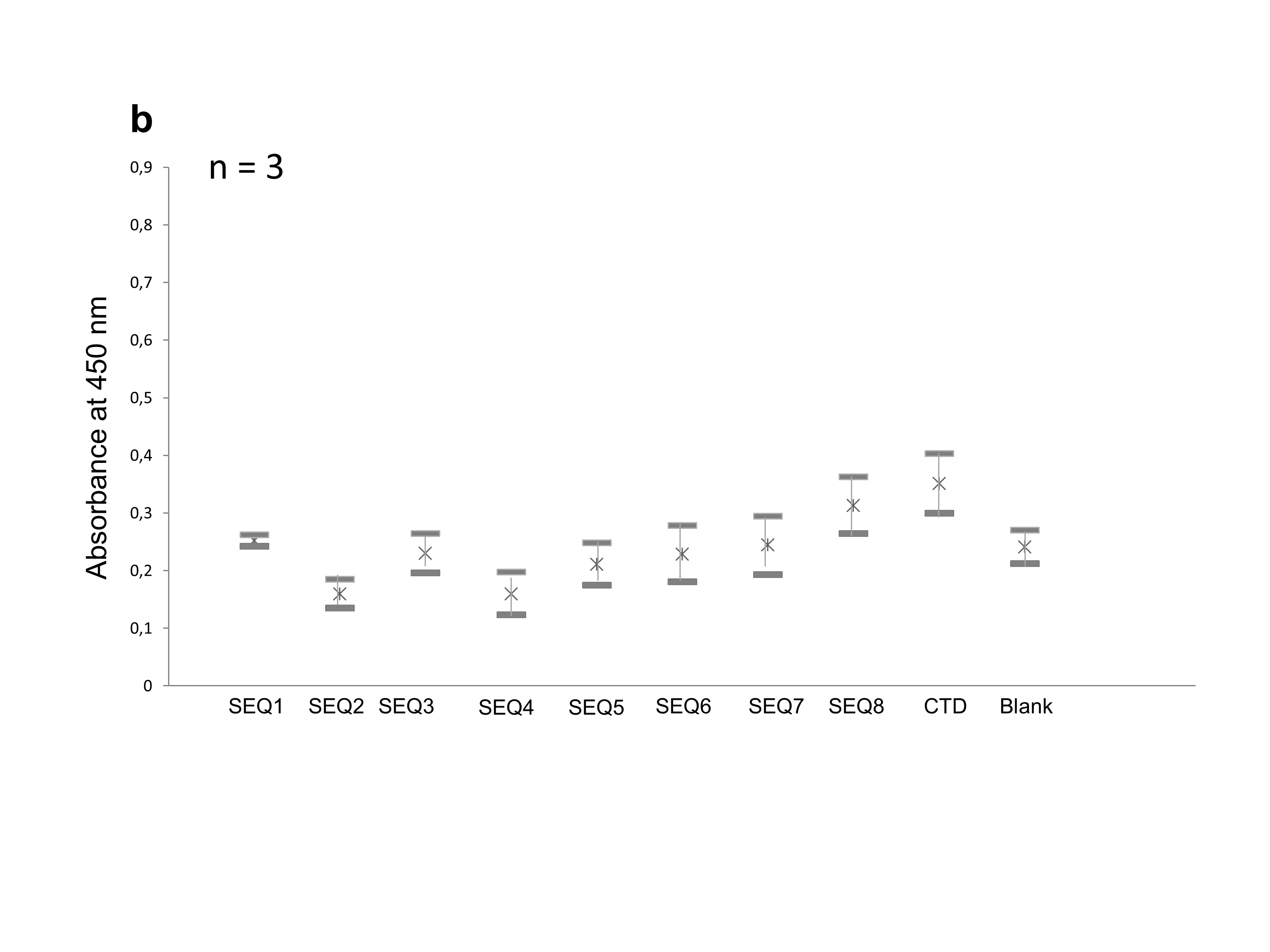


1. Buneva VN, Krasnorutskii MA, Nevinsky GA. *Biochemistry (Moscow).* **2013**, *78*: 127-143. [↑](#footnote-ref-2)
2. Reverberi R, Reverberi L. *Blood Transfus*. **2007**, *5*: 227-240. [↑](#footnote-ref-3)
3. Lejeune D, Delsaux N, Charloteaux B, Thomas A, Breasseur R. *Proteins*. **2005**, *61*: 258-271. [↑](#footnote-ref-4)
4. An, Y., Raju, R. K., Lu, T. & Wheeler, S. E. Aromatic interactions modulate the 5’-base selectivity of the DNA.binding antibody ED-10. *J. Phys. Chem*. **118**, 5653-5659 (2014). [↑](#footnote-ref-5)
5. Jørgensen, A. S., Gupta, P., Wengel, J. & Astakhova, I. K. ["Clickable" LNA/DNA probes for fluorescence sensing of nucleic acids and autoimmune antibodies](http://findresearcher.sdu.dk/portal/da/publications/clickable-lnadna-probes-for-fluorescence-sensing-of-nucleic-acids-and-autoimmune-antibodies(de616268-e76f-43be-86ac-6eb92fb88d05).html). *Chem. Commun*. **49**, 10751-10753 (2013). [↑](#footnote-ref-6)
